# Editorial for the Special Issue “Research on Performance Improvement of Advanced Alloys”

**DOI:** 10.3390/ma19112224

**Published:** 2026-05-25

**Authors:** Guozheng Quan, Chuntang Yu

**Affiliations:** 1 Chongqing Key Laboratory of Advanced Mold Intelligent Manufacturing, School of Materials Science and Engineering, Chongqing University, Chongqing 400044, China; 2 College of Materials Science and Engineering, Chongqing University of Technology, Chongqing 401320, China

## 1. Introduction and Scope

This Special Issue, “Research on Performance Improvement of Advanced Alloys”, provides an overview of recent research on the processing, microstructural regulation, surface and interface engineering, and service-performance evaluation of advanced alloy systems. The collected papers address a broad range of metallic materials, including titanium alloys, magnesium alloys, niobium-based coating systems, Cr-Mo-V hot-work die steels, cemented carbides, and Al-Si cast alloys.

Advanced alloys remain indispensable to modern manufacturing because they provide the structural and functional basis for applications requiring lightweight design, high-temperature resistance, corrosion and wear resistance, and long-term reliability. Contemporary alloy research has moved from isolated composition adjustment toward integrated process–structure–property–performance design, as reflected in integrated computational materials engineering, complex alloy design, high-temperature alloy development, titanium and light-alloy metallurgy, solid-state joining, surface engineering, and casting-quality control [1,2,3,4,5,6,7,8].

Against this background, the Special Issue highlights how alloy performance can be improved through coordinated control of composition, thermal history, deformation path, surface condition, defect population, and service environment. The topics covered include oxidation-resistant coatings for refractory alloys, phase transformation in metastable beta titanium alloys, friction stir welding of rare-earth magnesium alloys, hot deformation and thermomechanical fatigue of hot-work die steels, hot forming of thin-walled titanium structures, microstructure–property regulation in cemented carbides, galvanic corrosion of lightweight hybrid structures, cavitation and corrosion-wear protection of engine components, and porosity control in Al-Si casting alloys. Within this Special Issue, representative studies on multicomponent silicide coatings and hot-deformation/process modeling of Cr-Mo-V die steel further illustrate the integration of composition design, processing control, and service-oriented performance evaluation [9,10].

## 2. An Overview of the Published Articles

The eleven research articles included in this Special Issue can be viewed through several connected themes rather than as isolated case studies. The first theme is surface and interface engineering for harsh service environments. Contributions in this group address oxidation-resistant coating design for niobium alloys, galvanic corrosion in magnesium alloy–steel couples, cavitation resistance of surface-treated cylinder liners, and corrosion-wear behavior of friction pairs in methanol-fueled internal combustion engines. These studies collectively show that surface stability, interfacial compatibility, and environmental resistance are essential components of alloy-performance improvement.

The second theme concerns thermomechanical processing, phase transformation, and component-scale forming. The published work on the TB17 titanium alloy clarifies how cooling rate affects α-variant selection and microstructure evolution, while the study on TC4 titanium alloy thin-walled hyperbolic structures links forming instability, stress state, and process control. For Cr-Mo-V hot-work die steels, this Special Issue includes studies on hot-deformation constitutive modeling, processing maps, industrial-scale forging, and strain-controlled thermomechanical fatigue. Together, these papers demonstrate that reliable alloy design must couple microstructural mechanisms with realistic processing windows and service loading conditions.

The third theme is joining and microstructure–property regulation in lightweight and hard materials. The friction stir welding study on the Mg-Gd-Y-Zn-Mn alloy illustrates the importance of dynamic recrystallization, phase fragmentation, and multi-scale strengthening in solid-state joining of high-strength magnesium alloys. The work on ultrafine-grained WC-based cemented carbides highlights the combined effects of carbon content, sintering temperature, binder design, phase stability, hardness, and flexural strength. These contributions underline the need to balance strength, ductility, toughness, and structural integrity through coordinated processing and microstructural control.

The fourth theme relates to casting quality and defect control. The contribution on the AlSi9 alloy examines how iron content influences porosity and intermetallic formation in cast alloys intended for alfining piston-ring inserts. This work reinforces a broader conclusion of this Special Issue: performance improvement often starts with controlling defect formation during manufacturing, because pores, inclusions, brittle intermetallics, and local compositional heterogeneity can strongly affect reliability even when nominal alloy composition is acceptable.

Overall, the articles collected in this Special Issue show that advanced alloy performance is governed by the interaction of composition, processing, microstructure, surface condition, defects, and service environment. The value of these contributions lies not only in the specific alloy systems investigated, but also in their shared emphasis on establishing process–structure–property–performance relationships that can support both scientific understanding and engineering application.

## 3. Conclusions

The papers collected in this Special Issue show that the performance improvement of advanced alloys is a multidimensional task. Although the material systems are diverse, the contributions share a common scientific direction: the development of robust process–structure–property–performance relationships that can support alloy design, manufacturing optimization, and service-performance assessment.

Several cross-cutting trends can be identified. First, alloy-performance optimization is increasingly pursued through integrated frameworks rather than isolated empirical adjustment. Second, advanced characterization is often combined with constitutive modeling, processing maps, or service-performance evaluation to explain not only what occurs, but also why it occurs. Third, the research focus is moving from coupon-level material evaluation toward component-scale manufacturing, realistic service environments, industrial verification, and long-term reliability.

Important challenges remain. Future studies should further strengthen multi-scale predictive frameworks capable of linking thermal history, deformation paths, phase transformation, defect evolution, surface degradation, and long-term service performance. Greater attention should also be paid to coupled service factors, such as oxidation with thermal shock, corrosion with wear, and mechanical cycling with microstructural coarsening. In addition, process robustness, reproducibility, scale-up transferability, and sustainability should become more central considerations in advanced alloy design.

We hope that this Special Issue will be of interest to researchers and engineers working on alloy design, forming and joining, surface engineering, heat treatment, microstructural characterization, and service-performance assessment. The contributions presented here are expected to stimulate further integrated studies on advanced alloys and to support the development of materials and processes for increasingly demanding industrial applications.

## References

[1] National Research Council (2008). Integrated Computational Materials Engineering: A Transformational Discipline for Improved Competitiveness and National Security.

[2] Miracle D.B., Senkov O.N. (2017). A Critical Review of High Entropy Alloys and Related Concepts. Acta Mater..

[3] Pollock T.M., Tin S. (2006). Nickel-Based Superalloys for Advanced Turbine Engines: Chemistry, Microstructure, and Properties. J. Propuls. Power.

[4] Boyer R., Welsch G., Collings E.W. (1994). Materials Properties Handbook: Titanium Alloys.

[5] Polmear I., StJohn D., Nie J.F., Qian M. (2017). Light Alloys: Metallurgy of the Light Metals.

[6] Mishra R.S., Ma Z.Y. (2005). Friction Stir Welding and Processing. Mater. Sci. Eng. R Rep..

[7] Davis J.R. (2001). Surface Engineering for Corrosion and Wear Resistance.

[8] Viswanathan S., Apelian D., Donahue R.J., DasGupta B., Gywn M., Jorstad J.L., Monroe R.W., Sahoo M., Prucha T.E., Twarog D. (2008). ASM Handbook, Volume 15: Casting.

[9] Zhou X., Xiao L., Zha Y., Xu J., Fang J., Deng G., Xu S., Liu S., Zhao X., Cai Z. (2024). Study on the Oxidation Behavior of TiB_2_-CeO_2_-Modified (Nb,Mo,Cr,W)Si_2_ Coating on the Surface of Niobium Alloy. Materials.

[10] Yuan Y., Lin Y., Wang W., Zhang B., Shi R., Zhang Y., Xie J., Wu C., Mao F. (2024). Study on High-Temperature Constitutive Model and Plasticity of the Novel Cr-Mo-V Hot-Work Die Steel Forging. Materials.

